# An Experimental Inquiry into the Nature of Gravelly and Calculous Concretions in the Human Subject; and the Effects of Alkaline and Acid Substances on Them, in and out of the Body

**Published:** 1806-08

**Authors:** Thomas Egan


### *An experimental Inquiry into the Nature of Gravelly and Calculous Concretions in the Human Subject; and the Effects of Alkaline and Acid Substances on them, in and out of the Body.* By THOMAS EGAN, *M.D. M.R.I.A.*

THE constant occurrence of these afflicting complaints in Simpson's Gouty Hospital, to which I have been physician for several years, first turned my serious attention to the most probable means of alleviating or removing them. But to obtain this desirable end, an examination into the nature of the predisposing and proximate causes; of the chemical and other properties of gravelly matter itself; and that species of calculus most generally resulting from its aggregation, as well as of the remedies, and their mode of operation, became indispensibly necessary. I must also acknowledge, that I was not a little excited to this inquiry by the consideration, that, whilst the medicines now most confided in by modern practitioners are supposed to exert no energy on those substances out of the body, yet their beneficial effects, taken internally, stand uncontroverted by the experience of almost every physician.

Induced by these motives, I had, as far back as the year 1799, instituted a series of experiments, in hopes of throwing some more light on this subject; and, perhaps, che mically explaining upon what ground alkaline substances in general alleviate, whilst acids as constantly aggravate, this afflicting disease.

But, knowing that Messrs. Fourcroy and Vauquelin had been, for many years, particularly engaged in the analysis of urine and its morbid concretions; and expecting, from their superior abilities in researches of this kind, that the object which I had in view would be more satisfactorily fulfilled, I did not wish to intrude any observations of my own on the public.

After, however, most anxiously attending to the result of their scientific labours on this subject, as they have been, since that period, successively detailed by M. Fourcroy, in the *Annales de Chimie*, Memoirs of the National Institute, and in his great and elaborate work the *Connoissances Chimiques*; and finding little, if indeed any thing, illustrative of the subject, to which I would wish to point the attention of the faculty as well as the public in general, I again latterly repeated, with much care, my experiment of 1799, and added some more, which may probably prove interesting in a practical point of view.

These,

These, with some observations, and deductions from them, I now, with diffidence, offer to the candour and consideration of the academy.

I must here premise, that the limits of an academic dissertation necessarily confine me chiefly to the consideration of gravelly matter itself, and that species of calculus which most generally results from its aggregation.

Though determined to intrude as little as possible on their time by an useless quotation from antient authors, who could have no clear ideas of the subject; yet the better illustration of my object, as well as a sense of justice, oblige me to go as far back as Van Helmont, whose great though eccentric genius first observed that the subject matter of calculus existed in the urine itself. But the flighty extravagance of his ideas, of which he has given us a specimen on this subject in his Treatise de Lithiasi, (a wonderful production for the time) caused little attention to be paid to his opinion; and it was reserved for the capacious and learned genius of Boerhaave first to ascertain, beyond future doubt, the presence of gravelly matter as a natural constituent part of urine, kept in chemical solution in it, and eliminated by it out of the system. Of this important fact no material use was made, until the all-prying genius of the immortal Linnæus induced him to request his friend Scheele to turn, for a moment, his great chemical abilities to the investigation of this subject; with what success is but too well known. And from this again had arisen the farther prosecution of this inquiry by the celebrated Bergman.

The result of the analysis of the latter was highly honourable to the former chemist, as they perfectly agreed in almost every particular, with the exception of some small quantity of insoluble matter, and the presence of lime, observed by Bergman; a difference now very easily accounted for; the former having examined calculi of the pure lithic acid, or, as it is now termed, uric kind, (by far the most common species,) and entirely soluble in pure alkaline lixivia and nitric acid; the latter, those of the mixed kind, consisting also chiefly of lithic acid, but with interposed laminæ; or probably a nucleus of either calcareous phosphate or oxalate of lime, which frequently occurs in a very large proportion of these concretions. We may also observe, that Bergman had not, at this period, an adequate idea of the large proportion and insolubility of animal matter contained in them.

From their joint analysis it was, for the first time, proved proved that the subject matter of gravel, and of a very large proportion of calculi, was present in a state of real chemical solution in all healthy urine; that it was possessed of the following distinguishing chemical properties.

Insipid, inodorous, crystallizable, nearly insoluble in cold water, and only soluble in some thousand times its weight of boiling water; separable again from this, upon cooling, in a beautiful and peculiar crystalline form; of easy solubility in pure alkaline lixivia, which it renders sweetish, and neutralizes; precipitable from these again by the weakest acids, and still possessing its original crystalline form and properties. That, from these circumstances, with that of turning the vegetable blues red, it was of an acid nature, soluble in nitrous acid with effervescence ; this solution tinging the skin and other animal matters red, and, upon evaporation to dryness, assuming a red rose colour: this last property being peculiarly characteristic of this substance; subliming in part by distillation, without any alteration in its properties, and affording carbonate of ammonia, and other usual animal products, partly from the admixture of animal matter, and probably some adhering urea. To these distinguishing chemical properties of the Swedish chemist, Fourcroy has since added the following: When triturated with a lixivium of either of the fixed alkalies, it forms a matter of a saponaceous consistence, very soluble with excess of alkali, but little so without it. The saturated urates of potash and soda are little sapid, soluble, or crystallizable. By precipitating their dilute solution by muriatic acid we obtain the lithic acid in brilliant needle-like crystals, very voluminous, a little coloured, tending to the yellow, or *fauve,* as he calls it. Ammonia exerts little, if any, solvent power upon it: lime water takes up a little. The alkaline carbonates have no action upon it; and this last circumstance, I would beg leave to observe, has continued to be the opinion to this day; but how far founded, will appear in the sequel. To this matter Scheele gave the name of *lithic acid;* by which it continued to be known, until our countryman, Dr. Pearson, has latterly proposed that of *uric;* a change greedily adopted by the French chemists, as being more particularly indicative of its origin. In compliance with the philosophers of both nations, I shall, in future, term it *uric acid,* and the concretions of that nature, calculi of the uric-acid kind. The publication of Scheele's Essay excited the experimental inquiries of both chemists and physicians. His experiments were, accordingly, repeated peated by several of our countrymen in particular; but with various, and in many instances different, results.

It was already cursorily observed, that Bergman's analysis differed from Scheele's in some circumstances, which he, even at that period, was disposed to attribute to a difference in the nature of the calculi which they respectively examined; and this conjecture has been fully established by every subsequent inquiry since that time. We accordingly find a paper of Dr. Dawson's, in the London Medical Transactions for the year 1769, showing these concretions to be of very different and opposite kinds, and of course, soluble in very different and opposite kinds of menstrua: as also a letter from Dr. Saunders to Dr. Percival, of Manchester, published in the third volume of Percival's Philosophical and Experimental Essays, in 1776, detailing several experiments; from which he fairly concludes that the Doctor's enthusiastic hope, of dissolving all calculi in a solution of carbonic acid, must prove groundless, from the very different nature of their component parts, as ascertained by his own experiments. This was placed beyond further doubt by our own learned and ingenious Professor Mr. William Higgins, who, in an analysis of a calculus, of which he gives an account in his Comparative View of the Phlogistic and Antiphlogistic Theories, (a work of singular merit for that period, to which we will afterwards refer,) and published so far back as 1789, enumerates the many various substances contained in one specimen only. The researches of Austin, Lane, and Brugnatelli, led to similar results. But to the learned and accurate Dr. Wollaston we stand indebted for the first clear and distinct discrimination of the component parts of these substances. In a paper read to the Royal Society in the year 1797, which would not discredit either a Bergman or a Klaproth, he has most accurately demonstrated, both analytically and synthetically, the component parts of three distinct species of calculi; namely, the fusible, as he terms it, or the ammoniaco-magnesian phosphate of Fourcroy; the mulberry, or oxalate of lime kind; and bone earth calculus, or phosphate of lime, which, with the uric, well known to us since the time of Scheele, left us then acquainted with the four species of calculi of most frequent occurrence. Under these circumstances I cannot help expressing my surprise at finding M. Fourcroy still assuming the merit of the discovery of all the different component parts of calculi, the uric acid and phosphate of lime excepted. This circumstance must appear the more unaccountunaccountable, when we consider that the communication of Dr. Wollaston's experiments was through the medium of the Transactions of the Royal Society for 1797. Finally, M. Fourcroy, to whom Europe stands not a little indebted for the present general diffusion of chemical knowledge, and to whom the medical profession owe the greatest obligations for his unremitted application to animal chemistry, has, in conjunction with Vauquelin, given us the result of his researches upon five hundred calculi; from which it appears that they contain the seven following ingredients:

1. Uric acid.
2. Urate of ammonia.
3. Phosphate of lime.
4. Ammoniaco-magnesian phosphate.
5. Oxalate of lime.
6. Silica.
7. Animal matter.

From the prevalence of any of these ingedients, or their relative proportions, he divides them into four genera; and these again into twelve species; for an account of which I must refer to the tenth volume of the Connoissances Chimiques, and the Memoirs of the National Institute; not proposing to go into their chemical properties further than may be necessary to my present inquiry; namely, of how far acids may be conducive to the formation, or alkalescent substances to the prevention, or even solution, of a large proportion of gravelly and calculous concretions. We have already remarked, that to the sagacity of Boerhaave we are indebted for the knowledge of gravelly matter being a constituent part of urine kept in chemical solution in it; and, happily for mankind, only separable from it after being some considerable time out of the body. After minutely detailing the ingenious means made use of by Boerhaave to ascertain this important point, to which I beg leave to refer, his commentator, Van Swieten, goes on to observe:

"Hoc calculi rudimenta adsunt etiam in urina hominum sanissimorum; quæ, si una cum urina secernuntur, antequam ab urina secesserint, et concrescere inceperint, nullo modo sanitatem lædent. Cum autem observatum fuerit, illam separationem rudimentorum calculi citius fieri in quibusdam hominibus, tardius in aliis, patet, illos magis calculo obnoxios vivere, in quibus citius hæc separatio arenularum obtinet. An quandoque illa separatio contingit jam jam in renibus, et in vesica, antequam urina expellatur de corpore? Certe videtur. Vidi sæpius, una cum urina excretum sabulum nephriticum expulsum fuisse, statimque, calente adhuc et fumante urina, in fundo matulæ subsedisse. Contigit aliquoties, inventam fuisse, in linteis sanorum infantum urina madidis, copiam sabuli nephritici, satis duri, quod videtur una cum urina excretum fuisse. Cum enim magna cura haberetur, ne hi infantes, (illustri genere nati,) diutius urinâ, vel aliis sordibus, conspurcati et urina statim per lintea penetret, vix videtur possibile fuisse, ut in urina jam emissa hoc sabulum productum fuerit, intra unam alteramve horum."

And again he adds: "Hoc sabulum, in urina etiam sanissima concrescens, vocari posset calculus nativus; a quo nemo liber est; at qui tunc tantum metuendus videtur, si cito in urina concrescat. Felices illi, in quibus tardissime hoc fit. Propriam sæpius examinavi urinam, lætusque vidi, rudimenta illa prima calculi separari quam tardissime, requiri quandoque horas viginti quatuor et ultra, antequam in sabulum majoris molis concrescere potuerint. Sed et, licet decimum tertium ætatis lustrum emensus jam fuerim, ab omni lithiasi immunis vixi."

The mode and appearances attending the separation and crystallization of this substance from healthy urine, is one of the most beautiful that, probably, chemistry affords. But, as the circumstances are so minutely and correctly detailed by Boerhaave, and his commentator, Van Swieten, in his treatise *De Calculo*, vol. v. p. 201 and 202, and correspond so much with my own experiments, so often repeated, I must refer to him. On this passage, however, I must observe, that the space of twenty-four hours, mentioned by him as the period of spontaneous separation, is by far, in the healthy state, too short, and that it extends to two, three, and sometimes more days, according to the existing temperature and other circumstances. Nothing, therefore, I will presume to say, is more erroneous than the assertion, repeated in almost every chemical book, that the uric acid separates from urine upon cooling. When this occurs, which frequently happens, particularly with children, the urine is certainly surcharged with this very insoluble substance.

An increased temperature hastens the incipient decomposition of urine, and its first ammoniacal degeneration is always attended by the deposition of its uric acid in its crystalline form.

This

This did not escape the observation of Hales, who tells us, that urine, tending to putrefaction, affords most of this acid substance; and, indeed, were it to be deposited upon cooling, or within the space of twenty-four hours, or even more, as is so generally asserted, it should every day present itself to physicians, who so constantly attend to the state of urine in glasses; but this is by no means the case: and we find Fourcroy, in his last publication, mentioning from twenty-four to forty-eight hours, which certainly only applies to summer heat, or the circumstance already mentioned.

Our next great obligation is, undoubtedly, to Scheele, who has made us acquainted with its nature, and the very distinct chemical properties already enumerated.

While in the state of gravel it is ever the same, whether passed immediately with the urine, or spontaneously deposited, or precipitated from it; a circumstance that, for a long time, continued to give me much surprise, considering the variety of calculi; but of the truth of which I was convinced by the examination of many hundred specimens for many years back.

I was therefore pleased to find, that Fourcroy, for the first time, in his *Connoissances Chimiques*, asserts, "les sables des reins sont presque toujours de l'acide urique." And in another place he says, speaking of the uric acid, "c'est lui qui forme les sables, qui se crystallize, et s'attache aus parrois de vaisseaux."

No wonder, then, that calculi of this kind should be of most frequent occurrence; and that, of five hundred analysed by Fourcroy, one fourth should entirely consist of it, besides its occasional admixture with the remainder; and of three hundred, examined by Pearson, the greater number were found to be of this nature.

Having premised these necessary observations, we have now to consider to what circumstances we may attribute its separation, in a crystallized or aggregate state, from its natural solvent; the only condition in which it can be productive of inconvenience, or diseases of this kind. And first, I would observe that, being a natural secretion, of which the urine is only the vehicle destined to carry it out of the system, it must be subject to the same derangements with the other secretions of the human body, and may, of course, sometimes exceed in quantity, and at other times be more deficient; which last circumstance seems to take place during the continuance of acute diseases.

That

That a morbidly increased secretion does frequently occur, and that, too, independent of external causes, we have the most satisfactory proof of in the hereditary dispositions of many families to this complaint: and, indeed, when we consider the same to take place, relative to the functions and secretions of the liver, we must not be surprised at similar deviations in those of the kidneys. Here, truly, they are of more mischievous tendency, as, from the very sparing solubility of the uric acid, (even in its own natural menstruum, the smallest excess in quantity must subject it to precipitation.

Having premised these necessary considerations, I shall proceed to inquire into those circumstances which the experience and observation of all times have pointed out to us as the most frequent occasional causes of these maladies, and how far these opinions may be confirmed by experiments instituted for that purpose.

And first, it is a matter of notoriety, that the period of life, from infancy to about fifteen inclusive, is most subject to disorders of this kind.

Of this practical observation we have an interesting confirmation inserted in the second volume of the Memoirs of the French National Institute, Mathemetical and Physical Sciences, year 7. Under the former happy regime there was instituted, about forty years ago, at Luneville, in Loraine, an hospital for the exclusive relief of calculous and gravelly patients. In that interval, 1629, of both sexes, were admitted, and operated upon. Of these, 1564 were males, and only 65 females.

C. Saucerotte, an associate of the Institute, to whom we are indebted for these interesting details, annexes tables indicative of the number of these patients, that occurred at the different periods of life, from the age of one up to seventy-eight. To these, as too extensive to be inserted here, I would beg leave to refer; and shall satisfy myself with some extracts only, expressive of the general result.

| Age of Patients. Male Sex. | Number of Patients. |
|---|---|
| 1 year to 2 - - - | 1 |
| 2 years - - - | 14 |
| 3 - - - - | 79 |
| 4 - - - - | 131 |
| 5 - - - - | 145 |
| 6 - - - - | 147 |

From

From this age, which afforded the maximum of the number of patients, we find a gradual declension as follows:

| Age of Patients. | Number of Patients. |
|---|---|
| 8 years | 121 |
| 10 | 79 |
| 15 | 39 |
| 20 | 16 |
| 25 | 7 |
| 30 | 8 |
| 35 years | 4 |
| 50 | 5 |
| 60 | 2 |
| 70 | 2 |
| 78 | 1 |

Of the sixty-five females,

| Age of Patients. | Number of Patients. |
|---|---|
| 1 year to 3 | 1 |
| 4 | 8 |
| 5 | 7 |
| 6 | 4 |
| 7 | 6 |
| 8 | 5 |
| 9 | 3 |
| 12 | 4 |
| 14 | 1 |

From which period, down to seventy-eight, there occurs but one or two upon each year. From these, then, we learn how much more subject the male sex is to those complaints than the female; and the earlier periods of life than the more advanced. For among the males in the sixth year we find 147 (the greatest number), and among the females only five at eight. From these periods, in both sexes, the numbers rapidly diminish.

These facts would lead us to conclude that some physiological cause, peculiar to the functions of this early stage, may give rise to this difference; and I will not pretend to say but this may possibly exist: but when we consider that in every country the infant poor are the greatest sufferers, we are induced to inquire further, and suspect the existence of some general cause affecting and applicable to them all. That a similarity of diet (in the children of this class of society, in particular) must every where nearly take place, is evident; and that this is, but too often, of the kind most prone to the acescent tendency, such as pap, gruel, sour milk, &c.; all which it is not always in the power of the parents to renew, or administer, in a recent and sound state; an error not unfrequently occurring from the negligence of nurses even in the upper ranks, but irremediable in the lower; where this acescent tendency cannot be corrected by the seasonable admixture of broth, or other light animal food, their unhappy situation confining them exclusively, like their cattle, to the sole use of vegetables and the farinacea.

To pass on from infancy to the advanced periods of life, and begin with our own island, we find that, considering the extent of our population, the disease is of relative rare occurrence: so much so, that the late Mr. Dease, whose premature death we have still to deplore, as a national calamity,) with all his well deserved celebrity as a lithotomist, never operated upon more than sixty. A small number, indeed, when we consider that the operation is seldom, if ever, attempted in the country. And why this should happen here, we shall be presently, perhaps, better able to judge.

The reverse of this occurs in the sister kingdom; and the Irish student feels astonished at the frequency of the operation in all the London hospitals, though also performed in those of the more considerable country towns; and, upon inquiry, he finds that a large proportion of these patients come up from the cyder counties of Hereford, Devon, &c. and it must naturally occur to him, that the general use of fermented liquors of every kind, beer, cyder, perry, and factitious wines, which prevail in England, render the disease of more frequent occurrence there than with us, the great mass of our people being deprived of these luxuries.

If we pass over to the Continent, we find our neighbouring provinces, Picardy, Normandy, and Britany, in particular, still more subject to affections of this kind; so much so, that the late Mr. Dease could not give credit to the extraordinary number of patients operated on, in one year only, in the hospital of Rouen; though many must have, of course, repaired to Paris. The same, though in a lesser degree, takes place in Champagne; and it is almost unnecessary to observe, that the general beverage of the northern provinces consists of cyder, or of poor wine, equally acescent in its nature: and prone to the acetous fermentation. The Champagne, though somewhat less so, is replete with carbonic acid gas and disengaged tartarous acid; and though, in the more southern provinces, this malady malady cannot be considered as endemial, yet it is of frequent occurrence in the hospitals of Montpellier.

For, even in these favoured climes, where wine is of so little value, and withal so spirituous, the unfortunate peasant is obliged to content himself with an inferior quality prepared by a second maceration of the *marc* of the grape, which he denominates *picquet;* a *patois* appellation, most happily applied to its highly acid quality.

In that once happy country, Switzerland, on the contrary, as Baron Haller assures us, the disease is by no means frequent, and chiefly confined to the children of the poorer sort; their mountainous and elevated situations affording them little or no vinous liquors; whereas their neighbours, the inhabitants of the Rhine and Moselle, as well as some tracks on the banks of the Danube, are peculiarly afflicted.

The truth of this observation we find confirmed by the medical authors of all times. Silvius observes, "Vina acida tenuia et Rhenana, magis nocere calculosis quam opima;" and the same is particularly insisted on in Dolæus's "Encyclopædia Ephemerides Naturæ Curiosorum," and Rivinus's "Morbi Endemici," &c. Now, the wines in these countries are well known to be of an acid quality: and Hoffman asserts, and that too from experiments, that they abound in the tartarous acid, having found them to contain a double relative quantity of that in other wines; and to this we may add no small proportion of carbonic acid. Linnæus, in his dissertation "De Genesi Calculi," inserted in the second volume of the "Amœnitates Academicæ," seems more particularly to point out acids, and acescent drinks, as the chief causes of calculous affections. He says, "Acida fermentescentia omnia calculum promovent; hinc vina acida genesi calculi magis favent, quam dulcia. Qui acida vina copiose ingurgitant, podagræ et calculo plus exponuntur, quam illi, qui terras calidiores inhabitant, et dulcia vina hauriunt. Nec mirum, cum vini Rhenani libræ quatuor destillatione dant spiritus acidi drachmas quinque; et vini Tocariensis præbet spiritus acidi tantum semidrachmam, teste Hoffmanno. Sanissimus quisque a potu acido sæpe stranguriam incurrit, eo quod ab acidis ingestis particulæ terrestres præcipitantur." And again: "Quin podagræ igitur et calculus ab acido generentur, nullum est dubium, id etiam ab eorum communi cura, ad quam pergimus, luculentius patebit." Beverovie, *De Calculo*, 80, also observes: "In nullo vino tantum tartari apud nos accrescit, quam Rhenano. De me ipso, quod etiam ex plurimis audivisse memini, possum testari, nunquam Rhenanum assumsisse paulo largius, quin copiose arenulas excernerem."

The reverse of all this is observed to take place where the use of wine is prohibited. Rivinus observes, that in the city of Batavia, where the pursuit of commerce brings together a vast assemblage of the neighbouring Asiati nations, whenever the disease occurs, it is almost always in the instance of some Hollander, who, in his passage to India, drank freely of bottled beer, and used sour crout. In Persia, the same author, in his excellent treatise De Morbis Endemicis, observes, that whenever calculous affection occurs, either in Ispahan or the provinces, it is assuredly in the instance of some Armenian; fellows, (to use his word,) who, in every latitude, drink more wine than water.

Again, in Grand Cairo, where the proximity of the Grecian islands, and ready conveyance by the Nile, render wine of easy acquisition, and drunkenness and public houses as common as in any city in Germany; we learn, from Prosper Alpinus, that the disease is of very frequent occurrence; for, besides a mixed population of Franks, Armenians, Arabs, &c. the Mamelukes, as well as many other Turks of the higher ranks, do not, in deference to the Mahometan law, refrain from wine. The Cyprian and Grecian wines, if not adulterated, or become acescent by dilution, and the warm temperature of that city, are, in themselves, among the least objectionable. But, when we consider that Paris is chiefly supplied with Burgundy, and that yet in no part of the world does there occur more mischief from the attempts to keep down and correct its acescency, we shall easily form an opinion of the quality of the wine retailed in Cairo.

To this abstinence, then, from wine and fermented liquors; as also, perhaps, to the admixture of a large proportion of the warmest spices in their vegetable food, tending to correct its acescent tendency; we may ascribe the rare occurrence of this disease in the more southern climates.

Now, these more general remarks we find peculiarly to coincide with the observations of the patients themselves, as well as that of the physician; for such as have laboured under these complaints a sufficient length of time to become acquainted with the *juvantia* and *lædentia*, most scrupulously abstain from acids and acescent drinks of all kinds, and, what they find most particularly pernicious, beer beer or ales turning over to the acetous fermentation, or *hard*, as they are generally termed. And, indeed, nothing is more common, than that an indulgence in cyder, claret, or acidulated punch, nay a draught of hard beer or porter, should be followed by a fit of the gout and gravel.

The connection between these diseases forms an interesting and curious subject of physiological as well as pathological inquiry; but, proposing to offer some observations on this subject on a future occasion, I shall at present decline entering upon it, and pass on to observe, that the bad effects of all acidulous drinks are fully confirmed by the experience of our many sufferers in Simpson's hospital. Hewson, who lately died there at the advanced age of 102, never tasted the beer of the house during the summer months, and substituted milk for it; being taught by experience, that its acid tendency, during that period, always induced his gravelly paroxysms. And Clapham, who suffered much from gout and gravel, and was for many years a ship captain, informed me his voyages to America were always succeeded by fits of both; which he attributed to a free indulgence in the use of cyder, a beverage to which he was then peculiarly attached; and that, at any time, he could excite a paroxysm of one or the other, or both, by drinking acidulated punch, or claret. Khensk our greatest martyr (having all his articulations distorted by gouty concretions, and who once lived in easy circumstances,) assured me that the severest and longest protracted fit of the gout and gravel he ever experienced was occasioned by a surfeit of a poor vapid claret. And I shall conclude this part of my subject by observing, that the clergy of the Roman catholic church are peculiarly liable to these complaints, and form no small proportion of the number operated upon in this city; which I would attribute to the use of a small and sour wine during their residence in their seminaries abroad.

( To be continued. )

